# Editorial: Controversies and Perspectives in the Use of Postoperative Radiotherapy for Prostate Cancer

**DOI:** 10.3389/fonc.2017.00275

**Published:** 2017-11-23

**Authors:** Alan Dal Pra, Thomas Zilli, Stephane Supiot

**Affiliations:** ^1^Department of Radiation Oncology, Bern University Hospital, Bern, Switzerland; ^2^Department of Radiation Oncology, University of Miami Miller School of Medicine, Miami, FL, United States; ^3^Department of Radiation Oncology, Geneva University Hospital, Geneva, Switzerland; ^4^Department of Radiation Oncology, Institut de Cancérologie de l’Ouest, Nantes, France; ^5^Centre de Recherche en Cancérologie Nantes-Angers (CRCNA), UMR 892 INSERM—6299 CNRS, Institut de Recherche en Santé de l’Université de Nantes, Nantes, France

**Keywords:** prostate, radiotherapy, adjuvant, salvage radiotherapy, prostatectomy, prostate cancer

**Editorial on the Research Topic**

**Controversies and Perspectives in the Use of Postoperative Radiotherapy for Prostate Cancer**

The use of radical prostatectomy in patients with high risk of recurrence has significantly increased during the past 10 years (1). Thus, adjuvant radiation as a part of multimodality treatment or salvage radiation at the evidence of prostate-specific antigen (PSA) progression represents mainstay curative-intent options for a great number of prostate cancer patients. Although, few randomized trials and many retrospective studies have been published, many uncertainties still mold the discussions on the best treatment management for men after prostatectomy. This research topic (https://www.frontiersin.org/research-topics/3739/controversies-and-perspectives-in-the-use-of-postoperative-radiotherapy-for-prostate-cancer) successfully intended to foster discussions on current controversies in the use of postoperative radiotherapy and to present novel perspectives for treatment optimization.

Several randomized trials have shown that dose intensification in the primary treatment of prostate cancer improves local control. However, the data are scarcer in the postoperative setting. Beck et al. review the literature and present the only randomized phase III trial addressing dose-intensified salvage radiotherapy (64 vs. 70 Gy), SAKK (Swiss Group for Clinical Cancer Research) 09/10 (2). Recent publication showed that acute toxicity (gastrointestinal and urinary) and early quality of life data were not significantly different between the two treatment arms; however, a significant worsening of urinary quality of life was noted in the 70-Gy arm. The primary endpoint analysis (biochemical relapse free survival) and long-term endpoints are eagerly awaited.

Potential overtreatment and/or radiation-related toxicity with subsequent impact on patient’s quality of life are common arguments for withdrawing or deferring postoperative radiotherapy by urologists. By revealing gaps between evidence and clinical practice, Raziee et al. (Raziee and Berlin) claim that concerns with toxicities and/or quality of life should not preclude the utilization of curative-intent postoperative radiotherapy. Also, Herrera and Berthold review level I evidence on adjuvant radiotherapy that demonstrates improvements in biochemical progression-free survival, clinical progression-free survival, and overall survival in patients with high-risk pathological features (Herrera and Berthold). However, they point out that offering immediate adjuvant radiotherapy to all men with high-risk features would overtreat around 50% of men who would anyway be cancer-free, exposing them to unnecessary toxicity and adding important costs to the health-care system. The assessment of adjuvant versus early salvage radiation is being addressed in important randomized trials to be published in the forthcoming years (Radiotherapy and Androgen Deprivation in Combination After Local Surgery, Radiotherapy–Adjuvant versus Early Salvage, and Groupe d’ Étude des Tumeurs Uro-Génitales) [Raziee and Berlin; Herrera and Berthold].

The role of ADT in combination with primary radiotherapy for intermediate- and high-risk prostate cancer is well established. Recently, two prospective phase III trials (RTOG 9601 and GETUG-16) have shown improvements in disease outcomes when ADT is combined with salvage radiotherapy (3, 4). However, in the setting of early salvage, the role of ADT remains debatable. In patients with pre-SRT PSA <0.7 ng/ml, which comprised >50% of the RTOG 9601 study population, the addition of ADT provided no improvement in overall survival or metastasis-free survival. ADT is not devoid of important side effects, and many questions are still open on which patients benefit the most, ADT type, and treatment duration.

In parallel, the impact of the increasing aging population on the worldwide burden of cancer is well known, and the management of prostate cancer in the elderly is a topic of utmost importance. Goineau et al. specifically shed light on the care of elderly patients with prostate cancer after prostatectomy. The authors propose a decision tree based on the International Society of Geriatric Oncology recommendations.

Novel imaging modalities are reshaping the use of postoperative radiotherapy in prostate cancer patients. Molecular imaging has provided increasing accuracy in the localization of recurrence, and it has progressively changed clinical practice. Novel imaging tools can define the site of the recurrence and the extent of disease and thus individualize salvage treatments. In this research topic, Amzalag et al. comprehensively review most important novel targeted tracers for the evaluation of recurrent disease.

Analyses of large multi-institutional retrospective series along with predictive nomograms have importantly helped clinicians to estimate individual patient’s risks and tailor treatment decisions (5, 6). More lately, genomic classifiers have been added to the armamentarium of clinicopathological parameters and novel imaging modalities, representing an emerging tool able to provide exciting prognostic information for patients with recurrent disease (7–10). A better identification of patients with indolent and more aggressive tumors will help to select which patients may derive the greatest benefits from treatment intensification or deintensification and thus reducing therapy-associated costs and unnecessary adverse effects.

In terms of radiotherapy technique, important variability in the delineation of the prostate bed is observed. At least four international contouring consensus guidelines are available, but present discrepancies in target definition. This is a relevant topic when comparing outcome data from different retrospective and prospective cohorts. Latorzeff et al. from the GETUG group highlight some controversies to help clinicians create an appropriate volume delineation of the prostate bed in the setting of adjuvant and salvage radiotherapy (Latorzeff et al.). Also addressing variability in contour delineation, Delpon et al. critically report on automated atlas-based segmentation algorithms (Delpon et al.). The authors compare different commercially available options that could assist radiation oncologists in potentially improving contour delineation. Not to mention on the unclear benefits of elective treatment of the pelvic nodes which is currently addressed in the ongoing RTOG 0534 trial.

Image-guided radiotherapy is a key advancement in modern radiotherapy to decrease normal tissue toxicity. Vilotte et al. reviewed the literature on image guidance techniques in the postoperative setting (Vilotte et al.). The authors highlight key points on different techniques applicable to the prostatic bed and discuss potential reductions in planning target volume margins to reduce treatment complications.

By using an innovative approach for locally advanced tumors with high risk of local recurrence, Buge et al. present a preclinical evaluation of intraoperative low-energy photon radiotherapy using spherical applicators. With cadaveric models assessed by MRI, the authors show that intraoperative radiotherapy of the prostate bed is feasible, with good coverage of targeted tissues, and is potentially able to replace external beam radiotherapy in the future. Clinical studies are warranted to validate this exciting approach that could further decrease normal tissue toxicity.

Finally, in view of current and evolving data, the use of postoperative radiotherapy should be made in the context of a multidisciplinary discussion on treatment benefits and potential risk of side effects. Patients should take a proactive role in the decision-making process with unbiased, transparent, and evidence-based information. New imaging modalities and commercially available biomarkers have been increasingly utilized in the clinic, but unfortunately have not been timely incorporated into prospective studies. This dissonance between novel tools and lack of robust validation is a destiny not only in Radiation Oncology but also in other disciplines with rapidly evolving technologies. Hopefully, all this progress will ultimately lead to improvements in outcomes that matter most to our patients.

## Author Contributions

All the authors contributed either for initial writing and/or review of this editorial.

## Conflict of Interest Statement

The authors declare that the research was conducted in the absence of any commercial or financial relationships that could be construed as a potential conflict of interest. The reviewer, RT and handling editor declared their shared affiliation.
